# Mitochondria as a Target for Mitigating Sarcopenia

**DOI:** 10.3389/fphys.2018.01883

**Published:** 2019-01-10

**Authors:** Paul M. Coen, Robert V. Musci, J. Matthew Hinkley, Benjamin F. Miller

**Affiliations:** ^1^ Translational Research Institute for Metabolism and Diabetes, Florida Hospital, Orlando, FL, United States; ^2^ Department of Health and Exercise Science, Colorado State University, Fort Collins, CO, United States; ^3^ Aging and Metabolism Research Program, Oklahoma Medical Research Foundation, Oklahoma City, OK, United States

**Keywords:** skeletal muscle, aging, sarcopenia, exercise, mitochondria, treatment

## Abstract

Sarcopenia is the loss of muscle mass, strength, and physical function that is characteristic of aging. The progression of sarcopenia is gradual but may be accelerated by periods of muscle loss during physical inactivity secondary to illness or injury. The loss of mobility and independence and increased comorbidities associated with sarcopenia represent a major healthcare challenge for older adults. Mitochondrial dysfunction and impaired proteostatic mechanisms are important contributors to the complex etiology of sarcopenia. As such, interventions that target improving mitochondrial function and proteostatic maintenance could mitigate or treat sarcopenia. Exercise is currently the only effective option to treat sarcopenia and does so, in part, by improving mitochondrial energetics and protein turnover. Exercise interventions also serve as a discovery tool to identify molecular targets for development of alternative therapies to treat sarcopenia. In summary, we review the evidence linking mitochondria and proteostatic maintenance to sarcopenia and discuss the therapeutic potential of interventions addressing these two factors to mitigate sarcopenia.

## Introduction

The Centers for Disease Control (CDC) projects that the proportion of adults older than 65 years of age in the US population will grow from 12.4 to 19.6% reflecting the aging baby boom generation and declining birth rates in the United States. Furthermore, the population of persons older than 80 years is expected to more than double from 9.3 million in 2000 to 19.5 million in 2030 (Centers for Disease Control and Prevention (CDC), 2003). This aging phenomenon is not unique to the United States since the number of individuals aged 65 years and older is expected to nearly double from 6.9% of the world population in 2000 to 12.0% by 2030 (Beard et al., 2016). Revisions to such predictions even suggest that by 2050, the population of those over the age 65 in the United States will be up to 108 million, 25.8% of the predicted population (Olshansky et al., 2009).

Since the aged population is increasing globally, the prevalence of sarcopenia, the age-related loss of skeletal muscle mass and function, is likely to increase as well. Global prevalence of sarcopenia is difficult to measure in part due to changing consensus of what constitutes the diagnosis of sarcopenia. In 1998, Baumgartner et al. defined sarcopenia as the age-associated loss of skeletal muscle mass two standard deviations below a healthy population (Baumgartner et al., 1998). Based strictly on skeletal muscle mass loss, Baumgartner and colleagues estimated that 24% of individuals less than 70 years of age have sarcopenia while 50% of those over 80 years of age had sarcopenia (Baumgartner et al., 1998). More recent analyses find widely discrepant disease incidence with NHANES data collected between 1999 and 2004 reporting that 27.8 and 19.3% of men and women at least 60 years of age were sarcopenic (Janssen et al., 2002). Other estimates are as high as 35.4 and 52.5% for women over 60 and 80 years of age, respectively, and 75.5 and 88.1% for men over 60 and 80 years of age (Batsis et al., 2014). Surprisingly, there is a paucity of data that detail the economic burden of sarcopenia, although an analysis over a decade old found that the healthcare cost of sarcopenia in the United States was an estimated $18.5 billion per year (Janssen et al., 2004).

Skeletal muscle is the largest organ in the human body and plays a key role in posture and capacity for locomotion, as well as serving as a bona fide endocrine organ (Pedersen and Febbraio, 2012). As such, skeletal muscle dysfunction has detrimental effects on many aspects of human health for older adults. Epidemiological studies have found that sarcopenia increases the overall risk for mortality (Landi et al., 2013; Batsis et al., 2014). In part, this is because sarcopenia increases the risk of developing mobility disabilities, leading to impairment in activities of daily living by twofold (Janssen et al., 2002), and risk of falls by three times (Landi et al., 2013). The loss of muscle mass and function is not exclusive to postural/locomotor muscle groups, as myopathy of key inspiratory muscles also occurs with aging, resulting in respiratory failure (Kelley and Ferreira, 2017). The increased risk of disability from respiratory failure has led to greater hospitalization of older adults (Verissimo et al., 2015; Kelley and Ferreira, 2017). The combined effect of sarcopenia and hospitalization further exacerbates muscle dysfunction, as older adults do not adequately recover from bed rest (Suetta et al., 2009, 2013; Hvid et al., 2010), which contributes to reduced functionality and ambulation upon discharge, and leads to loss of independence, nursing home placement, and increased risks of falls (Fortinsky et al., 1999; Mithal et al., 2013). In addition to physical disability, sarcopenia contributes to the development of cardiovascular and metabolic diseases because of its involvement in substrate metabolism (Batsis et al., 2015) and as an endocrine organ (Pedersen and Febbraio, 2012).

The current consensus is that to diagnose sarcopenia, one should assess walking speed or grip strength and then examine appendicular lean mass if either is below a certain cutoff value (Cruz-Jentoft et al., 2010; Fielding et al., 2011; Morley et al., 2014). If muscle mass is lower than a healthy population cutoff value, sarcopenia is diagnosed. As such, low muscle mass continues to be an important component in the definition of sarcopenia. In 2016, the World Health Organization established an ICD-10 code for sarcopenia, which will spur the development of effective therapeutic strategies and increase the recognition of the importance of maintaining muscle mass and function with age for overall human health (Anker et al., 2016).

**Figure 1:**
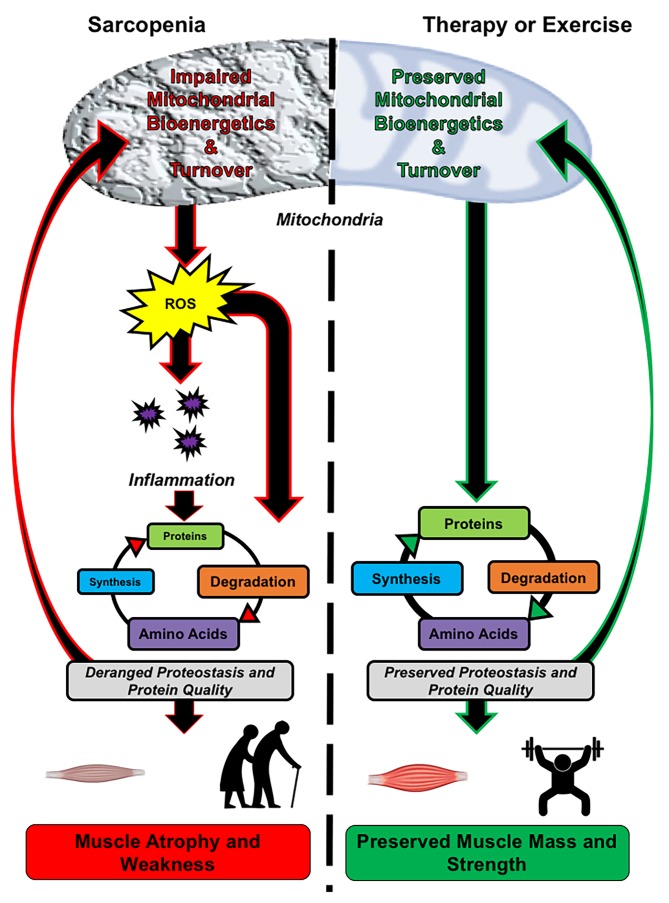
Role of mitochondrial bioenergetics and proteostasis in mediating skeletal muscle quality in older adults. *Left panel,* Sarcopenia is associated with mitochondrial dysfunction, which encompasses impaired bioenergetics and turnover. The impairment results in increased reactive oxygen species (ROS) generation and chronic low-grade inflammation, leading to impaired muscle proteostasis. The derangement in proteostasis impedes mitochondrial turnover, resulting in an accumulation of dysfunctional mitochondria and further exacerbation of organelle and tissue dysfunction. *Right panel,* Targeting mitochondrial bioenergetics and turnover by therapeutics and exercise impedes the age-associated rise in ROS and systemic inflammation, which results in the maintenance of muscle proteostasis. The maintained protein turnover allows for the removal of damaged proteins, such as dysfunctional mitochondria and damaged contractile proteins, while also synthesizing new functional proteins. Collectively, this leads to preservation of mitochondrial quality, muscle mass, and strength.

Resistance exercise continues to be the most effective intervention against sarcopenia (Landi et al., 2014). In addition, maintenance of physical activity can delay the progression of sarcopenia (Power et al., 2016a; Lazarus and Harridge, 2017). Despite the strong support for maintaining an active lifestyle, adherence to physical activity guidelines remains low. The traditional therapeutic focus of sarcopenia treatment is to target growth-related pathways to increase muscle mass. Here, we discuss the positives of these strategies, but also build a case for targeting mitochondrial bioenergetics as a way to maintain muscle mass and function with age as summarized in Figure 1. This review will cover the etiology of muscle loss, three basic characteristics of aging that may contribute to sarcopenia, current treatments targeting mass, and how targeting mitochondria rather than mass could mitigate basic mechanisms of aging to slow sarcopenia.

## Mechanisms Leading to Loss of Strength and Function

### Etiology of Muscle Loss

The etiology of sarcopenia is characterized by both slow, gradual loss of muscle mass over time that is propagated by acute periods of accelerated loss and poor nutrition (English and Paddon-Jones, 2010). Acute periods of muscle loss in older individuals is often met with an incomplete regain of muscle mass and strength, thus accelerating gradual sarcopenic progression (Suetta et al., 2009; White et al., 2015; Baehr et al., 2016). The inability to completely regain muscle mass and strength is common in both aging humans (Suetta et al., 2009) and animals (White et al., 2015; Baehr et al., 2016). This public health problem is particularly troublesome for older adults who comprise the majority of hospital patients in the United States (DeFrances et al., 2008; Fisher et al., 2010) and who may lose more muscle mass during bed rest (Paddon-Jones et al., 2004; Kortebein et al., 2007). Older adults do not adequately recover following bed rest without adequate rehabilitation (Suetta et al., 2009, 2013; Hvid et al., 2010), which likely contributes to their reduced functional status and ambulation upon discharge (Covinsky et al., 2003; Coker et al., 2015). It is important to target both the gradual and accelerated periods of muscle loss to mitigate the progression of sarcopenia.

The vast majority of adults fail to meet physical activity guidelines. While 60% of adults, both European and American, self-report that they meet guidelines, objectively measured physical activity reveals that fewer than 10% of adults in the United States meet physical activity guidelines (Tucker et al., 2011; Marques et al., 2015). Moreover, sedentary behavior alone increases the risk for sarcopenia. While there are few trials in humans on the effects of lifelong sedentary behavior, studies in mice reveal lifelong sedentary behavior impairs mitochondrial function (Figueiredo et al., 2009). Moreover, a cross-sectional study in men revealed that sedentary behavior increased inflammation independent of physical activity (Parsons et al., 2017). Other lifestyle behaviors such as diet, combined with a sedentary lifestyle, also predispose individuals to increased risk for sarcopenia. An analysis of four prospective studies revealed that obesity increased the risk of developing sarcopenia by 20–162% (Stenholm et al., 2008). Indeed, sarcopenia and obesity often occur together, and evidence suggests that risk of metabolic disease and mortality increase when both are present (Wannamethee and Atkins, 2015). Obesity is characterized by inflammation and insulin resistance, both of which contribute to vascular dysfunction, as indicated by reduced endothelium-mediated vasodilation, impaired skeletal muscle perfusion, and decreased myofiber capillary density. Greater adipose infiltration into skeletal muscle is associated with the loss of skeletal muscle strength and torque (Goodpaster et al., 2001a). Lifestyle factors contribute to changes in the basic processes that lead to muscle loss.

Muscle mass is controlled by a dynamic balance of protein synthesis and degradation. A loss of muscle mass occurs when protein synthesis and degradation tip toward net degradation. Strategies to maintain muscle mass have focused on increasing protein synthesis since this is thought to be the more dynamic regulator of mass. Signaling through the mechanistic target of rapamycin (mTOR) pathway is the major regulator of protein synthesis in skeletal muscle (Bodine et al., 2001; Glass, 2005). The activation of mTOR results in activation of p70 S6K causing an increase in protein translation, and the inhibition of 4e-binding protein (4e-BP1), which is a negative regulator of the eukaryotic translation initiation factor-4e (EIF-4e). The activation of these pathways stimulates growth processes, primarily of the myofibrillar proteins. However, a focus purely on growth may not maintain protein quality as discussed later in the review.

### Mechanisms of Muscle Loss—Basic Processes

#### Loss of Proteostasis

Proteostasis is the maintenance of protein homeostasis that refers to the location, concentration, conformation, and turnover of individual proteins (Balch et al., 2008). Proteostasis is essential for whole body and tissue function. Loss of proteostasis leads to the accumulation of damaged proteins (Levine and Stadtman, 2001; Ayyadevara et al., 2016). In skeletal muscle, impaired proteostasis could lead to the decline in quantity and quality of contractile proteins because of accumulation of damage and non-enzymatic modifications of these proteins. One such non-enzymatic modification of proteins, advanced glycation end-products (AGEs) (Semba et al., 2010) increases in concentration with age including in skeletal muscle (Haus et al., 2007). Because of cross-bridge formations, modified proteins are resistant to breakdown and accumulate and may contribute to tissue dysfunction (Barreiro and Hussain, 2010; Drenth et al., 2016) and mobility disability (Sun et al., 2012).

Since the enzymatic capacity to repair protein damage is low, protein turnover is essential to maintain the skeletal muscle proteome. Increased protein turnover should improve proteostasis by degrading damaged proteins and resynthesizing new, functional proteins. Therefore, even in the absence of muscle growth, protein turnover is a beneficial adaptation for tissue health. Protein turnover is an energetically costly process with a large proportion of this demand coming from the step of translation. Protein synthesis requires 12–72 ATP molecules per amino acid synthesized, an additional four phosphates (either ATP or GTP) per bond, and an additional 1–2 ATPs per fold (Lynch and Marinov, 2015). The energetic cost of protein breakdown remains only partially understood. Protein degradation occurs primarily through protein ubiquitination and proteasome degradation, or *via* the autophagy-lysosomal system. Ubiquitination requires 2 ATPs per ubiquitin tag and subsequent proteasome-mediated degradation of them requires between 100 and 200 ATPs per protein (Lynch and Marinov, 2015). Lysosomal degradation requires four ATPs for every amino acid. Altogether, protein synthesis accounts for 20% of basal metabolism and protein breakdown comprises another 5–15% of basal metabolism (Rolfe and Brown, 1997). Given the energetic demands of protein turnover, a reliable source of energetic production is essential to maintain proteostasis.

#### Reactive Oxygen Species

The balance between oxidant production and scavenging is essential in maintaining redox homeostasis. With age, redox homeostasis declines and leads to the progressive oxidation of cellular components, including contractile proteins, which leads to skeletal muscle dysfunction (Lourenço dos Santos et al., 2015). Evidence from studies of human skeletal muscle indicates a positive correlation between age and markers of oxidative damage including lipid peroxidation, protein carbonyl content, and 8-oxo-deoxyguanosine (8-oxo-dG), a measure of DNA oxidation (Capel et al., 2005).

Age-related impairments in mitochondrial function result in increased production of reactive oxygen species (ROS), which increases oxidative stress and contributes to the loss of proteostasis. Mitochondrial dysfunction contributes to an imbalance of reducing equivalents such as NADH, which increases ROS formation and leads to the oxidation of cellular components. Loss of structural integrity of mitochondrial supercomplexes and membranes also leads to increased ROS production and associates with impaired skeletal muscle function (Genova and Lenaz, 2014). An increasingly oxidized environment not only increases oxidative damage to cellular components, it also increases the formation of AGEs. In models such as the whole body SOD1 KO mouse (Jang et al., 2010) and the muscle-specific SOD1 KO mouse (Sakellariou et al., 2018), there is an accelerated muscle aging phenotype that resembles muscle aging in humans. Conversely, targeting oxidative stress has also shown efficacy at preventing the sarcopenic phenotype in aged mice (Lee et al., 2010; Vays et al., 2014). Therefore, these models provide support for an indirect and direct role of oxidative stress in skeletal muscle aging.

#### Inflammation

Inflammation is a response to a cellular disruption (infection, damage, detection of “non-self”) in an effort to facilitate a return to homeostasis. Chronic, low-grade inflammation occurs with aging due to immunosenescence and has been coined “inflammaging.” Inflammaging contributes to chronic diseases including cardiovascular disease and sarcopenia (Ferrucci and Fabbri, 2018). Pro-inflammatory cytokines including interleukin-1 (IL-1), interleukin-6 (IL-6), c-reactive protein (CRP), and tumor necrosis factor (TNF) are characteristic of inflammaging and therefore have been proposed as markers of age and disease risk (Justice et al., 2018).

Several studies have associated circulating inflammatory cytokines with decreased skeletal muscle strength. The InCHIANTI study, a prospective study of mobility in older adults, demonstrated that elevated inflammatory markers predicted handgrip and leg strength (Schrager et al., 2007). A Danish cross-sectional study indicated that inflammatory marker TNF was inversely related with lean mass (Pedersen et al., 2003). Others have found that high IL-6 (>5pg/ml) and CRP (>6.1pg/ml) levels increased the risk of losing more than 40% of muscle strength by twofold to threefold (Schaap et al., 2006). Given the evidence from corroborating studies of aging, it is likely that inflammation contributes to the etiology of sarcopenia (Chung et al., 2009).

The mechanisms by which inflammation contributes to sarcopenia are not completely understood. In a study of older male subjects, increased levels of pro-inflammatory ceramides impaired anabolic signaling following an acute bout of resistance exercise (Rivas et al., 2012) meaning that inflammation may impair normal anabolic responses. In addition, high-fat diet (HFD)-fed rodents and muscle cell culture models consistently link ceramide with exacerbated muscle atrophy or impaired anabolic signaling (Hyde et al., 2005; Roseno et al., 2015). There is also speculation that inflammatory factors such as prostaglandins impair skeletal muscle function by stimulating the generation of reactive oxygen species (ROS) (Chung et al., 2009). As an example, sphingomyelinase, an enzyme involved in the inflammatory response, stimulates the release of ROS that results in decreased contractile function as a consequence of chronic inflammation (Ferreira et al., 2010). More studies are needed to determine the mechanisms by which inflammation contributes to sarcopenia.

### Interface of Mitochondria and Basic Aging Processes

The decline in skeletal muscle mitochondrial capacity with aging has been extensively studied as a contributor to slower walking speed (Coen et al., 2013; Santanasto et al., 2016; Gonzalez-Freire et al., 2018), fatigability (Santanasto et al., 2015), and sarcopenia (Joseph et al., 2012; Gouspillou et al., 2014). Evidence from the Baltimore Longitudinal Study of Aging indicates that skeletal muscle *ex vivo* mitochondrial respiration parallels decline *in vivo* oxidative capacity, cardiorespiratory fitness, and muscle strength (Gonzalez-Freire et al., 2018). While a number of cross-sectional human studies have demonstrated lower mitochondrial function with chronological age (Trounce et al., 1989; Boffoli et al., 1994; Tonkonogi et al., 2003; Short et al., 2005; Lanza et al., 2008; Porter et al., 2015a), several others have failed to observe these changes (Rasmussen et al., 2003; Hutter et al., 2007; Larsen et al., 2012b; Gouspillou et al., 2014; Gram et al., 2014). These inconsistent results may be partially due to variation in the approaches used to assess mitochondrial function. In addition, many studies of mitochondrial function in aging have not controlled for important covariates including participant physical activity levels (Boffoli et al., 1994) and adiposity (Short et al., 2005), which likely confound the relationship between mitochondrial capacity and age (Proctor et al., 1995; Hutter et al., 2007; Lanza et al., 2008; Safdar et al., 2010). For example, Distefano and colleagues demonstrated a strong, inverse correlation between age and mitochondrial function (Distefano et al., 2016). However, when controlling for fitness and adiposity, age only accounted for 1–6% of the variation observed in maximal ATP production. Regardless, there is some factor associated with the aging process that contributes to mitochondrial decline. The National Institute on Aging (NIA) recently funded the study of muscle, mobility, and aging (SOMMA), the goal of which is to determine the combination of muscle properties (energetics, autophagy, denervation, and oxidative stress) that most strongly predicts major mobility disability, declines in fitness, 400-m walking speed, and muscle mass.

Evidence from preclinical models indicates a close link between mitochondrial energetics and control of muscle mass. Release of pro-apoptotic factors (Max, 1972; Adhihetty et al., 2007), morphological alterations (fission, swelling), energy stress *via* reduced ATP (Romanello et al., 2010), and increased mitochondrial reactive oxygen species (ROS) emission (Adhihetty et al., 2007; Müller et al., 2007; Kavazis et al., 2009; Min et al., 2011) have all been reported during muscle atrophy in preclinical studies. In addition, for a given concentration of ADP, mitochondria from aged muscle generate more ROS than young counterparts (Holloway et al., 2018). Combined with the age-related decline in endogenous antioxidant activity, the increase in ROS emission leads to an increase in concentration of unscavenged ROS (Dai et al., 2014).

Mitochondrial ROS can depress protein synthesis by decreasing phosphorylation of 4e-BP1 and impairing mTOR assembly (Pham et al., 2000; Shenton et al., 2006; Zhang et al., 2009). Mitochondria-targeted antioxidant treatment in rodents supports a crucial role for mitochondrial ROS in mediating muscle atrophy (Min et al., 2011; Powers et al., 2011). Increased ROS production can also exacerbate muscle atrophy (Jang et al., 2010). Mitochondrial ROS stimulate proteolytic degradation pathways (autophagy and proteasome system) (Li et al., 2003; Aucello et al., 2009; McClung et al., 2009; Hussain et al., 2010) and energetic stress (reduced ATP production), which can activate the AMP kinase (AMPK)-FoxO3 pathways leading to increased expression of the ubiquitin-proteasome system and lysosome-autophagy system (Greer et al., 2007; Romanello et al., 2010). Taken together, multiple lines of compelling preclinical evidence implicate a central role for mitochondrial energetics in muscle atrophy. In addition, ROS react with and damage cellular components, including contractile proteins, which decreases proteome integrity and increases the demand for somatic maintenance.

In regard to inflammation, dysfunctional mitochondria exacerbate the detrimental effects of pro-inflammatory cytokines. Mitochondria release damage-associated molecular patterns (mito-DAMPs) that stimulate inflammatory pathways (Tezze et al., 2017). The mito-DAMPs further act on proteins such as NOD-like receptor protein 3 (NLRP3), which is involved in heart disease and aging (Salminen et al., 2012). Along with increased ROS release and pro-inflammatory signaling, dysfunctional mitochondria exacerbate inflammatory signaling that impair cellular integrity and proteostasis, which consequently leads to myocyte death (Chung et al., 2009).

Mitochondria are central to maintaining the energetic resources to preserve proteostasis. When there is a mismatch between the rate of ATP production and the demand for ATP, expendable cellular processes are sacrificed (Hou, 2013). There are three broad categories of cellular processes: metabolism, growth, and somatic maintenance (Hou et al., 2008). Processes that fall under metabolism are those that sustain life including energy production. Growth involves cellular expansion (Gregory, 2001), while somatic maintenance involves maintaining the quality of the soma (Shanley and Kirkwood, 2000). If the ability to provide ATP on demand is constrained, the cell makes trade-offs between growth and somatic maintenance since metabolism is maintained (Hou, 2013). Thus, impairment of mitochondrial function can in turn compromise proteostasis. In addition, it is conceivable, although never tested, that a focus on growth processes could compromise cellular maintenance if not matched by increased energy-producing capabilities.

To illustrate the importance of maintaining mitochondria to mitigate the detrimental declines of basic cellular processes in sarcopenia, the neuromuscular junction (NMJ) provides an excellent example. Degradation of the NMJ leads to the denervation of muscle fibers (Jang and Van Remmen, 2011), which appears to contribute to decreased muscle size, strength, and endurance (Sundberg et al., 2018). With age, type II myofibers are more prone to denervation and are reinnervated with type I motor units (Kelly et al., 2017). Reinnervation of type I motor units leads to previously denervated type II myofiber to adopt a type I myofiber phenotype (Kelly et al., 2017). The change in innervation could contribute to the decline in skeletal muscle strength with age (Nilwik et al., 2013), as evident by the observation that older individuals have a greater proportion of type I myofibers along with lower strength (Frontera et al., 2000). Some evidence suggests that declines in NMJ quality precede the impairment of skeletal muscle function (Spendiff et al., 2016); however, more research is still needed in this area.

Oxidative damage of the NMJ promotes the loss of skeletal muscle proteostasis (Vasilaki et al., 2017). Recent data suggest that impaired redox signaling, rather than oxidative damage *per se*, drives denervation (McDonagh et al., 2016). Mitochondria in neurons of aged organisms appear to emit more ROS which can disrupt redox signaling by desensitizing redox sensors that are responsible for adaptation (McDonagh et al., 2016). With aging, there is decrease in the ability to activate redox-related signaling pathways, and this failure causes further oxidative damage in the neuron which promotes NMJ degradation (Vasilaki et al., 2006). Therefore, targeting mitochondrial function in the NMJ or areas surrounding the NMJ may be a mechanism to protect against age-related muscle loss.

## Non-Exercise Treatments of Sarcopenia

Treating sarcopenia has revolved around therapeutic interventions to mitigate the loss of muscle mass. These therapies, which include targeting members of the transforming growth factor β (TGF-β) superfamily, testosterone, selective androgen receptor modulators (SARMS), and growth hormone (GH), among others, are currently or have been tested at various phase clinical trials (Garber, 2016; Morley, 2016). In the following sections, we will discuss the mechanism in which these therapies target muscle loss, along with their efficacy to treat sarcopenia.

Growth and differentiation factor 8 (GDF8 or Myostatin), a member of the TGF-β superfamily, and activin A are powerful negative regulators of skeletal muscle growth (Lee, 2004). Myostatin and activin A signal through the activin type II receptor (ActRIIB), which leads to activation of Smad 2/3 transcription factors, translocation to the nucleus, and activation of target genes. Myostatin negatively regulates Akt signaling preventing protein synthesis and also interferes with myoblast differentiation into myotubes and may also impair muscle growth. A number of anti-myostatin antibodies have been developed and tested in humans, all of which increase lean mass (Becker et al., 2015; Woodhouse et al., 2016; Rooks et al., 2017) and some also showed improved physical function and strength (Becker et al., 2015; Rooks et al., 2017). It was recently demonstrated that inhibition of activin A in primates enhanced muscle growth (Latres et al., 2017). However, concerns remain around the functional benefits from inhibiting signaling through ActRIIB as in myostatin knockout mice, there is a loss of specific force (Amthor et al., 2007). Inhibiting myostatin has also been linked with reduced mitochondrial capacity of skeletal muscle, poor muscle endurance, and fatigability (Mouisel et al., 2014).

Low levels of circulating testosterone in older men (otherwise known as hypogonadism) are associated with reduced lean body mass, bone mineral density, and increased fat mass (Saad et al., 2017). Mechanisms of action for testosterone include increasing protein synthesis (Wolfe et al., 2000; Ferrando et al., 2003) *via* Akt/mTOR activation (White et al., 2013) and reduction in adipose stem cells and activation of satellite cell recruitment (Kovacheva et al., 2010). There is strong evidence from intervention studies that treatment with testosterone is effective in increasing lean mass and reducing fat mass (Kenny et al., 2010; Srinivas-Shankar et al., 2010; Kvorning et al., 2013). However, the efficacy of testosterone to improve muscle-specific strength and physical function is less clear (Snyder et al., 1999; Saad et al., 2017). The Testosterone Trial in Older Men showed that a year of testosterone treatment in 790 older men had no benefit with respect to fatigue or walking distance (Snyder et al., 2016). Conversely, others have shown that treatment with testosterone improved muscle function (leg extension, triceps extension, biceps curl) (Ferrando et al., 2002) and grip strength (Morley et al., 1993; Sih et al., 1997) in older men with hypogonadism. Whether testosterone influences skeletal muscle mitochondrial energetics is also not clear. Testosterone deficiency is associated with reduced myocellular metabolism and mitochondrial energetics (Traish et al., 2011) and preclinical work indicates that testosterone can induce mitochondrial biogenic signaling (Usui et al., 2014). However, testosterone treatment did not alter the activity of enzymes known to regulate mitochondrial biogenesis or markers of oxidative phosphorylation and lipid metabolism in the skeletal muscle of aging men with low testosterone (Petersson et al., 2014).

Growth hormone (GH) was also considered a therapeutic for sarcopenia. There is well-described decline in activity of the GH/insulin-like growth factor-1 (IGF-1) axis in older adults (Zadik et al., 1985). In 1990, it was shown that administration of human GH to older healthy individuals could increase lean mass and it was thought at the time that GH might be an effective antiaging therapy (Rudman et al., 1990). However, subsequent studies indicated that GH increased muscle mass but not strength (Kim and Morley, 2005) and unfavorable side effects were also reported, including gynecomastia and carpal tunnel syndrome, and treated patients were more likely to experience impaired fasting glucose (Liu et al., 2007). In a cross-sectional study of healthy adults, GH/IGF-1 level corresponded to muscle mitochondrial function (by ^31^P MRS) (Makimura et al., 2011). However, 2 weeks of GH treatment led to a reduction in mitochondrial genes involved in β-oxidation and oxidative phosphorylation (Sjögren et al., 2007). Many of the pharmacological targets explored to date are generally efficacious in improving muscle mass. However, and perhaps germane to the lack of therapeutic effectiveness, it is less clear whether mitochondrial energetics are improved with these options.

Manipulation of diet is another approach for the treatment of sarcopenia that has received a lot of attention. Post-feeding hyperaminoacidemia is a potent stimulator of skeletal muscle protein synthesis in young adults and is blunted in older adults following ingestion of amino acid mixture, suggesting skeletal muscle of older individuals is anabolic resistant (Volpi et al., 2000). It has been proposed that a greater dietary intake of essential amino acids could help older adults maintain skeletal muscle mass (Volpi et al., 2013). However, recent studies examining increased protein intake in older individuals do not show increased lean body mass (LBM) accretion (Bhasin et al., 2018), and that the amount and quality of protein intake are not associated with muscle mass or strength in older adults (Gingrich et al., 2017). Therefore, there remain questions over whether anabolic responses from protein and amino acid intake that are classically determined over the short-term translate to long-term preservation or improvements in muscle mass.

Increasing vitamin D levels, which is commonly reduced in older adults, through dietary supplementation results in improved muscle mass and chair-stand test time, a surrogate of muscle power in older adults (Bauer et al., 2015). Interestingly, improving the vitamin D status of adults that are deficient resulted in improved mitochondrial oxidative function in skeletal muscle (Sinha et al., 2013; Rana et al., 2014). This finding suggests that improvements in muscle health by vitamin D supplementation could at least be partly due to improvements in mitochondrial oxidative phosphorylation in older adults. A greater understanding of the independent roles of these dietary components on the regulation of muscle mass and function is needed along with a focus on how they also impact mitochondrial energetics.

As illustrated, despite substantial evidence linking mitochondrial energetics to the etiology of sarcopenia, pharmacological therapies to date have not focused on mitochondrial targets but rather pathways that mediate increases in muscle mass. There are some exceptions. For example, there is currently an ongoing clinical trial testing whether metformin, an AMPK activator, enhances the response to resistance exercise (Long et al., 2017). This proposed treatment has the potential to improve cellular metabolism and mitochondrial biogenesis to improve anabolic responsiveness. Other studies have examined the effects of exogenous antioxidants, such as vitamin C, on muscle quality and oxidative stress. Vitamin C supplementation improves muscle function in at least one model of aged muscle (Ryan et al., 2010), but vitamin C is known to inhibit the redox signaling that leads to positive mitochondrial responses (Gomez-Cabrera et al., 2008; Paulsen et al., 2014; Bruns et al., 2018). Meta-analyses investigating the efficacy of long-term vitamin C supplementation conclude that supplementation actually increases the risk for disease and mortality (Bjelakovic et al., 2012). Other strategies targeting antioxidant mechanisms are promising though. For example, studies using a purported Nrf2 activator that increases endogenous antioxidants produces lifespan in male mice (Strong et al., 2016) and enhances protein synthesis during aerobic exercise training (Bruns et al., 2018).

In sum, clinical therapies that use pharmaceutical and nutritional intervention that focus solely on muscle mass regulation have been met with limited success in various phase clinical trials. Additionally, these therapies appear to have little or no influence on mitochondrial energetics. Therefore, additional interventions are needed that help preserve mitochondrial function and muscle proteostasis in older adults. On the other hand, exercise has been shown to be an effective countermeasure to age-related muscle loss. Below, we focus on how exercise training affects mitochondrial function to support the notion that targeting mitochondrial function is essential in developing effective treatments to mitigate sarcopenia.

## Exercise for the Treatment of Sarcopenia

### Physical Activity With Aging

Physical activity tends to decline with aging and results in difficulties in daily life activities and normal functioning (Westerterp, 2000). Data from the Health, Aging, and Body Composition (HABC) study showed that older adults who maintain higher levels of physical activity levels are protected against functional or mobility limitations in comparison to sedentary adults (Brach et al., 2004). There are several mechanisms in which maintaining physical activity throughout the lifespan protects against the onset of sarcopenia. Physical activity is a determinant of mitochondrial function, which likely is one mechanism that maintains skeletal muscle function (Larsen et al., 2012a; Tevald et al., 2014). Compared to sedentary counterparts, lifelong and masters athletes maintain skeletal muscle function concomitantly with mitochondrial function (Zampieri et al., 2014; Power et al., 2016b). Physical activity also attenuates the age-related increase in inflammation and oxidative stress, which, as highlighted, contribute to the loss of skeletal muscle mass and function (Mikkelsen et al., 2013).

Physical activity in mid-life appears to have a protective effect on physical function, as Patel et al. showed that older adults who engaged in higher levels of physical activity in mid-life performed better on the Short Physical Performance Battery (SPPB) and have reduced incidences of mobility disability than those less active in mid-life (Patel et al., 2006). Collectively, these results reveal a potential effect of exercise pre-habilitation to prevent the deleterious effects of aging and sedentary lifestyle on muscle function in older adults. The pre-habilitative effects of physical activity have led to clinical investigation into whether interventions later in life can prevent muscle dysfunction with aging. The Lifestyle Interventions and Independence for Elders (LIFE) study was designed to determine whether a physical activity intervention might confer significant physical benefits in older adults in comparison to those in a health education program. The intervention included ~150 min/wk of moderate-intensity walking, along with strength, flexibility, and balance training (Fielding et al., 2011; Pahor et al., 2014). The 6-month intervention reduced major mobility disability in older adults that participated in the physical activity protocol, as well as a reduced risk of persistent mobility disability in comparison to those in a health education program (Pahor et al., 2014). Collectively, the results of the LIFE study concluded that moderate increases in physical activity levels, which likely improve cellular bioenergetics, blunt the detrimental effects of primary aging on physical function in older adults. In the following sections, we will describe how structured exercise interventions of different modalities (i.e., strength and aerobic exercise training) lead to improvements in skeletal muscle mitochondrial health in older adults.

### Resistance Exercise

Resistance exercise remains a widely prescribed means to prevent, mitigate, and even reverse sarcopenia. The efficacy of resistance exercise to stimulate lean mass accrual and increases in strength is well documented (Koopman and van Loon, 2009). It was thought that resistance exercise training had little or no effect on mitochondrial biogenesis or function. However, recent studies have shown that resistance exercise training increases mitochondrial protein fractional synthesis rates (FSRs) (Wilkinson et al., 2008; Robinson et al., 2017) and improves mitochondrial function (Porter et al., 2015b; Robinson et al., 2017). Young adults engaged in a resistance exercise program showed increases in mitochondrial enzyme activity and respiration (Porter et al., 2015b). While the changes in mitochondrial respiration are modest in comparison to endurance exercise, improvements in *in vivo* PCr recovery rates and oxidative capacity appear comparable in older adults engaged in either exercise intervention (Jubrias et al., 2001). Similarly, using permeabilized myofibers, resistance exercise training increases state III and maximal oxidative phosphorylation capacity in older adults, which is accompanied by improvements in ADP sensitivity (Holloway et al., 2018). Therefore, while resistance exercise directly stimulates myofibrillar protein accrual to promote strength, it may also have positive effects on mitochondrial function and proteostasis.

Novel training regimens, such as power training, have been proposed to improve muscle function in older adults. Power training has received significant interest to prevent the sarcopenic phenotype, due to its targeting of type II myofibers, which are more prone to atrophy in older adults (Purves-Smith et al., 2014; St-Jean Pelletier et al., 2017). Indeed, clinical studies have shown that power training improves muscle function in older adults that is comparable or slightly greater than traditional resistance exercise training (Reid et al., 2008; Tschopp et al., 2011). Though preservation of mitochondrial function through physical activity is an important factor in the prevention of sarcopenia, the effects of power training on organelle bioenergetics is unclear.

While resistance exercise remains an effective intervention to combat sarcopenia, there are several considerations when prescribing resistance exercise training to older individuals. These concerns have led to alternative resistance training programs that focus on low load. It appears that low-load, high-repetition exercise is equally effective as high-load exercise in stimulating muscle hypertrophy in healthy individuals (Schoenfeld et al., 2017). Blood flow restricted resistance exercise is a second modification that may reduce the load necessary for positive adaptations. The premise is that a metabolic stress (blood flow restriction) may stimulate both mitochondrial and hypertrophy responses. Even with low load and number of repetitions, blood flow restricted exercise can positively influence motor unit activation and hypertrophy (Farup et al., 2015). More studies on blood flow restriction are necessary to determine if there are additional mitochondrial adaptations beyond traditional resistance exercise.

### Aerobic Exercise

Aerobic exercise is generally not appreciated as a stimulator of hypertrophy; however, there is evidence that it can lead to muscle hypertrophy. In older adults, aerobic exercise training improves myofiber size and strength, as well as whole muscle size and strength (Harber et al., 2012). Therefore, aerobic exercise stimulates muscle growth either directly or indirectly.

Nearly half a century ago, Holloszy first documented that aerobic exercise increases mitochondrial content (Holloszy, 1967). Since then, research has consistently documented that aerobic exercise improves both mitochondrial content and function (Menshikova et al., 2006; Jacobs and Lundby, 2013; Zampieri et al., 2014). Aerobic exercise increases mitochondrial turnover since it increases both mitochondrial biogenesis (protein synthesis) (Wilkinson et al., 2008; Scalzo et al., 2014) and mitophagy (mitochondrial-specific autophagy) (Drake et al., 2015). The improvement in the rate of ATP production from aerobic exercise training suggests that more energy is available to maintain proteostasis. Additionally, improvement in mitochondrial efficiency (reduction in ROS generated per oxygen consumed or ATP generated) suggests that there is less oxidative stress and damage, which would in turn improve the quality of the proteome. In all, aerobic exercise mediated improvements in mitochondrial function likely protects against sarcopenia (Musci et al., 2017).

Enhanced mitochondrial content and function can sustain greater energetic flux and oxidation of substrates. Increased energetic flux can decrease the accumulation of lipotoxic intermediates that promote inflammation and oxidative stress (Goodpaster et al., 2001b; Coen and Goodpaster, 2012). Exercise-induced improvements in substrate flux thus decrease inflammation and oxidatively modified proteins (Corcoran et al., 2007; Fabbri et al., 2016). It has been shown that long-term aerobic exercise training in diabetic individuals can return mitochondrial function to that of a lean individual (Konopka et al., 2015). Importantly, aerobic exercise improves the ability of the muscle to provide energy on demand. Having energy on demand reduces compromises between growth and somatic maintenance that comes during periods of energy shortage. Thus, aerobic exercise improves the ability to maintain cellular integrity and adaptation to stress (Ristow and Schmeisser, 2014).

## Exercise as a Discovery Tool (MOTRPAC)

Exercise and physical activity enhance various aspects of human health and prevent metabolic and neurological diseases as well as cancer. In 2016, the National Institutes of Health (NIH) common fund dedicated $170 million to the molecular transducers of physical activity consortium (MoTrPAC). The goal is to identify the molecular networks in key tissues that are changed in response to acute and chronic exercise (Neufer et al., 2015). Elucidating how acute molecular responses to exercise integrate over time and improve health outcomes will unveil novel mechanisms contributing to health and disease processes and identify potential new therapeutic targets to aid in the prevention and treatment of disease. Older adults will be included in the study cohort and so the molecular networks that are altered in muscle with aging will be captured, in addition to the effects of exercise on mitochondrial and proteostasis molecular networks. From this study, there is enormous potential for us to further understand how endurance and resistance exercise improve mitochondrial function and muscle health in older adults.

## Future Directions

Additional studies are needed to directly answer the question of whether treatments that target mitochondrial adaptations, such as aerobic exercise, are sufficient to maintain muscle mass with age. From these studies, the formulation of practical guidelines that refine recommendations of frequency, intensity, and duration of such activities should be derived. After establishing the appropriate evidence, it will be important to integrate important modulating factors such as medications (Robinson et al., 2009) and dietary factors (Smith et al., 2015) for long-term outcomes. Although there is strong preclinical and clinical support for targeting mitochondria with exercise, more studies are needed to directly support its efficacy to mitigate sarcopenia.

## Summary

If one considers a few of the primary underlying contributors to the loss of muscle mass with age, that is, loss of proteostasis, inflammation, and oxidative stress, it is apparent that mitochondria should be a target to mitigate sarcopenia (Figure 1). Unfortunately, to date, the field has primarily focused on directly stimulating growth processes rather than basic processes that create an environment that is favorable for growth processes. In other tissue types such as cardiac, hepatic, and nervous, it is well recognized that maintaining cellular energetics is of primary importance for maintaining tissue quality with age. Although skeletal muscle mitochondria are viewed as important for metabolism, the role of mitochondria in maintaining muscle mass is less appreciated. In our opinion, targeting mitochondria with exercise or other treatments is an effective way to treat sarcopenia and one that is still underexplored.

## Author Contributions

BM conceived of the review. PC, RM, MH, and BM wrote the review. PC and MH created the figure. PC, RM, MH, and BM proofread the review.

### Conflict of Interest Statement

The authors declare that the research was conducted in the absence of any commercial or financial relationships that could be construed as a potential conflict of interest.
